# Current Advancements in Bone Grafting Substitutes for Osteoporotic Distal Tibia Fractures: A Narrative Review of Beta-Tricalcium Phosphate (Neobone™) and Demineralized Bone Matrix

**DOI:** 10.3390/medicina62071229

**Published:** 2026-06-25

**Authors:** Gab-Lae Kim, Nah Yon Kim, Young Yi

**Affiliations:** 1 Department of Orthopaedic Surgery, Kang-Dong Sacred Heart Hospital, Hallym University Medical Center, Gil-dong, Seoul 134-701, Republic of Korea; 2 Department of Orthopaedic Surgery, Ewha University Mokdong Hospital, Seoul 07985, Republic of Korea

**Keywords:** distal tibia fracture, osteoporosis, β-tricalcium phosphate, demineralized bone matrix, bone graft substitute, metaphyseal bone defect

## Abstract

The surgical management of distal tibia fractures in the elderly is increasingly complex due to the rising prevalence of osteoporosis and the unique anatomical constraints of the region. While autologous bone graft remains the gold standard, its limitations have led to the widespread adoption of synthetic and biological substitutes. This narrative review explores the current trends in bone grafting for osteoporotic distal tibia fractures, focusing on pure β-tricalcium phosphate (β-TCP) and demineralized bone matrix (DBM). We specifically examine the biological mechanisms, resorption kinetics, and clinical outcomes of these materials. Furthermore, we highlight the emerging clinical preference for powder-type pure β-TCP (Neobone™) due to its superior surface area and packing efficiency in irregular metaphyseal voids. Powder-type pure β-TCP demonstrates superior packing efficiency and predictable resorption kinetics for metaphyseal void filling, while DBM remains a context-dependent biological supplement. Ion-substituted TCP formulations, pharmacological augmentation, and hybrid scaffolds are highlighted as future directions.

## 1. Introduction

The global aging population has precipitated a sharp increase in fragility fractures, with over 178 million fractures occurring worldwide in 2019 alone [1]. Osteoporosis affects an estimated 200 million people globally, with fracture risk rising steeply after the age of 50 [2,3]. Distal tibia fractures, though less prevalent than hip or vertebral fractures, carry a disproportionate clinical burden due to their complex soft tissue environment, high rates of wound complications, infection, malunion, and non-union, and the significant functional impairment they impose on elderly patients [3,4,5]. Osteoporosis further compromises the mechanical stability of this region, which already possesses a vulnerable soft tissue envelope and precarious vascular supply [4,6]. Traditional internal fixation often fails to provide sufficient stability in these patients, necessitating the use of bone graft substitutes to fill voids created by metaphyseal impaction and to stimulate biological healing [3,4].

Among the available options, pure β-tricalcium phosphate (β-TCP) and demineralized bone matrix (DBM) have emerged as the primary choices for clinicians [7]. β-TCP offers a predictable, resorbable osteoconductive scaffold, while DBM provides essential osteoinductive growth factors [8]. However, the physical form of these substitutes—ranging from blocks and granules to fine powders—profoundly affects their clinical efficacy [9]. To our knowledge, no prior review has specifically addressed the integrated clinical context of osteoporosis, the anatomical challenges of the distal tibia, and the practical implications of material form selection. This review therefore aims to provide a focused synthesis and a practical decision-making framework for the treating orthopedic surgeon.

A narrative literature search was conducted using PubMed, Scopus, and the Cochrane Library, with search terms including ‘distal tibia fracture’, ‘osteoporosis’, ‘beta-tricalcium phosphate’, ‘demineralized bone matrix’, and ‘bone graft substitute’. Studies were selected based on relevance to the biological mechanisms, clinical outcomes, and material properties discussed in this review.

## 2. Anatomical and Pathophysiological Challenges

The distal tibia is a uniquely difficult site for fracture healing. Anatomically, the region has minimal muscular coverage, with approximately one-third of the bone circumference being subcutaneous [10]. This leads to a baseline of poor vascularity, which is further compromised during trauma. In the context of osteoporosis, the trabecular bone in the metaphysis becomes sparse, and the cortical shell thins, often leading to “die-punch”-type fragments and significant cavitary defects during a fracture [11].

Wolff’s Law dictates that bone remodeling is driven by mechanical stress, but in the osteoporotic environment, this relationship is dysregulated, leading to a biological milieu that is slow to repair and prone to non-union [2,12,13]. The primary goal of bone grafting in this region is to restore the structural integrity of the subchondral bone and provide a bridge for host cell migration [14,15,16].

Biomechanically, the distal tibia transmits the full axial load of the lower extremity to the ankle joint, making structural integrity of the subchondral bone critical for functional recovery. The surrounding structures—including the peroneal tendons, the anterior tibial neurovascular bundle, and the thin anteromedial skin—provide limited soft tissue coverage, increasing the risk of wound complications following surgical fixation [10]. In the osteoporotic setting, impaired osteoblast activity, reduced angiogenic response, and dysregulated inflammatory signaling further compound these challenges, with non-union reported in up to 10% of operatively managed distal tibia fractures [11,13].

## 3. Pure Beta-Tricalcium Phosphate (β-TCP): Characteristics and Mechanisms

Pure β-TCP (${Ca}_{3}{\left({PO}_{4}\right)}_{2}$) is an inorganic bioceramic with a calcium-to-phosphorus ratio of 1.5, closely mimicking the mineral phase of human bone [7,17]. Its primary mode of action is osteoconduction, providing a 3D scaffold for the “creeping substitution” process, where host bone gradually replaces the synthetic material [18,19,20].

Tricalcium phosphate (${Ca}_{3}{\left({PO}_{4}\right)}_{2}$) exists in two polymorphic forms: β-TCP and the more reactive α-TCP, which exhibits higher solubility and faster osseointegration kinetics. However, this accelerated resorption can outpace new bone formation, making the more predictable resorption profile of β-TCP preferable for metaphyseal void filling in the osteoporotic setting.

A critical factor in β-TCP’s success is its interconnected porosity. Clinical-grade β-TCP is typically engineered with macropores (250–400 μm) to allow for neovascularization and micropores that facilitate the adsorption of endogenous growth factors. Unlike non-resorbable hydroxyapatite, β-TCP undergoes cell-mediated resorption by osteoclasts, a process that is ideally synchronized with the formation of new bone by osteoblasts (Table 1).

Clinical studies have validated β-TCP as a reliable bone graft substitute. Hernigou et al. reported a 100% fusion rate with β-TCP versus 94% with autograft in open-wedge HTO, with less pain and fewer complications [21]. The bone remodeling process follows a well-characterized sequence of osteoclast-mediated scaffold resorption and osteoblast-driven new bone formation—‘creeping substitution’—with complete remodeling observed in approximately 55% of cases within 12 months [14].

**Table 1:** Key biological and structural properties of pure β-tricalcium phosphate and their clinical implications.

Feature	Pure β-TCP Property	Clinical Benefit
Resorption	Osteoclast-mediated (6–24 mo)	Replaced by native bone
Porosity	60–80% (interconnected)	Facilitates vascular ingrowth
Purity	≥95% phase pure	High biocompatibility; no inflammation
Osteoconduction	Superior to HA and α-TCP	Faster integration in healthy bone [22]

## 4. Demineralized Bone Matrix (DBM): Biological Potential

DBM is a biological substitute derived from allograft bone via acid extraction, which removes the mineral components while preserving the Type I collagen and endogenous bone morphogenetic proteins (BMPs) [23,24]. Its defining advantage is osteoinductivity—the ability to actively trigger the differentiation of mesenchymal stem cells into osteoblasts [18,25].

In the distal tibia, DBM is frequently used as an “extender” for autograft or as a biological stimulant in non-union cases [26,27]. While it lacks structural strength, modern formulations, such as those utilizing 100% human allograft with no extrinsic carriers, offer improved handling and the ability to be hydrated with bioactive fluids like platelet-rich plasma (PRP) [28,29].

Clinically, Walter et al. (2025) [26] reported satisfactory osseous union in 85.6% of foot and ankle cases treated with DBM; however, non-union remained the most common complication at 11.5%, and overall evidence quality was low with significant heterogeneity [30]. Kulachote et al. (2016) demonstrated significantly shorter healing time with DBM augmentation in atypical subtrochanteric femoral fractures (28.1 vs. 57.9 weeks, *p* = 0.04), suggesting benefit in cases with compromised healing potential [25]. These findings indicate that DBM’s clinical efficacy is context-dependent and requires careful patient selection.

## 5. Clinical Outcomes and Comparative Efficacy

Available evidence, largely from observational studies and non-inferiority trials, suggests that β-TCP may provide comparable structural outcomes to autologous bone graft with a potentially reduced operative burden; however, high-quality randomized controlled trial data specific to osteoporotic distal tibia fractures remain limited [7,21]. The rate of secondary joint surface collapse is actually lower in some β-TCP cohorts compared to biological substitutes, likely due to the material’s ability to maintain its volume during the early phases of remodeling [4,17] (Table 2).

## 6. Discussion: The Superiority of Powder-Type Pure β-TCP

A significant trend in modern orthopedic trauma is the shift toward using powder-type or fine granular formulations of pure β-TCP (e.g., Neobone™) for metaphyseal void filling. It should be noted, however, that the majority of supporting evidence derives from observational studies and non-inferiority trials, and that high-quality randomized controlled data specific to osteoporotic distal tibia fractures remain lacking. The following discussion therefore reflects current clinical trends and biological rationale rather than definitive evidence of superiority. Several biomechanical and biological factors support this preference:

### 6.1. Enhanced Surface Area and Resorption Kinetics

The physical form of the graft substitute determines its interaction with the physiological microenvironment. Powder-type pure β-TCP offers a vastly increased surface-area-to-volume ratio compared to large blocks or coarse granules [31]. This increased surface area accelerates the chemical dissolution and release of $Ca^{2+}$ and $PO_{4}^{-3}$ ions, which are essential for the precipitation of a carbonated hydroxyapatite layer that initiates cell attachment. This leads to a more rapid and uniform resorption profile, ensuring that the graft is integrated into the healing bone without leaving significant voids or persistent foreign material [32].

### 6.2. Packing Efficiency in Irregular Cavitary Defects

**Figure 1:**
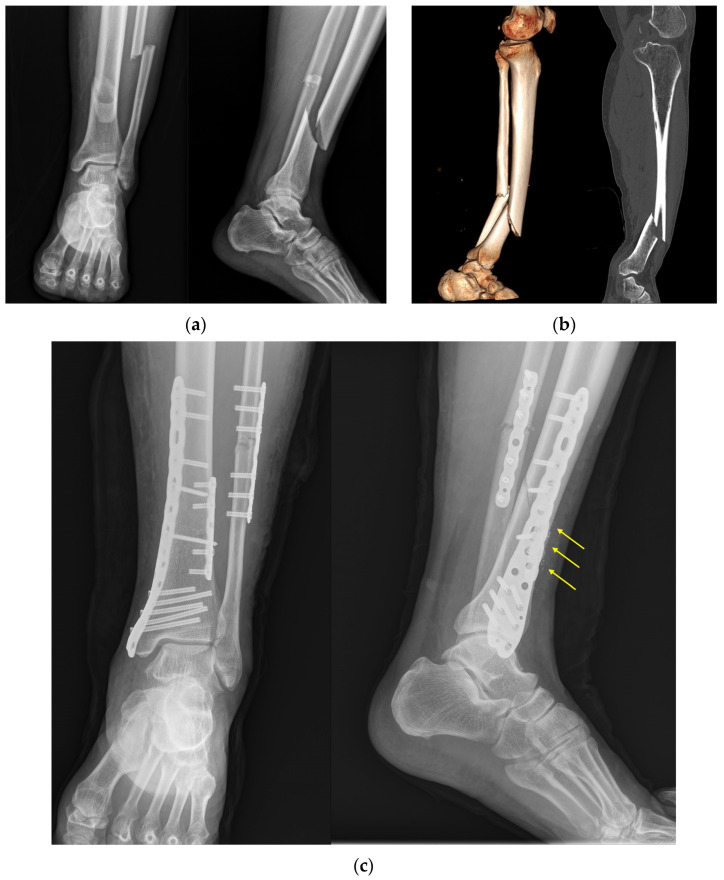
A 73-year-old man with a distal tibial fracture following trauma. (**a**) Preoperative radiographs demonstrating a distal tibial fracture with metaphyseal involvement and associated fibular fracture. (**b**) Preoperative CT images illustrating the fracture morphology and displacement. (**c**) Postoperative radiographs showing internal fixation and filling of the metaphyseal region using powder-type pure β-TCP (Neobone™) (arrows). Written informed consent for publication was obtained from the patient.

Osteoporotic distal tibia fractures often result in multi-cavitary, irregular defects that are difficult to fill with pre-shaped blocks. Powder-type β-TCP allows for superior packing efficiency, as the fine particles can be compressed into every crevice of the defect, ensuring intimate contact with the host’s endosteal surface. This “form-fitting” capability is crucial for providing immediate biological support to the subchondral bone and preventing the micro-movements that can lead to secondary collapse (Figure 1).

A recent preclinical study comparing putty and granular β-TCP in sheep tibial defects reported faster resorption and healing response with putty formulations; however, a significant decrease in osteoblast numbers was observed at 8 weeks in the putty group, suggesting that excessively rapid resorption may compromise synchronized bone remodeling [33]. Furthermore, putty formulations incorporate polymer binders that may introduce additional biological variables. Powder-type pure β-TCP avoids these concerns while maintaining superior surface area and form-fitting capability.

### 6.3. Microporosity and Growth Factor Storage

Recent research indicates that the intrinsic micropores within powder-based fillers can act as a reservoir for growth factors and bioactive peptides from the surrounding fluids. By sintering pure β-TCP powders into highly porous scaffolds, manufacturers can create a delivery system that slowly releases these biological cues, further enhancing the osteoinductive potential of an inherently osteoconductive material. In clinical studies of high-purity β-TCP, complete resorption and bone remodeling have been observed in approximately 55% of cases within 12 months, a rate that is considered optimal for metaphyseal healing [14].

## 7. Recent Trends: Bioactive Glass and Hybrid Materials

The “third generation” of bone grafts includes materials that are both bioactive and antimicrobial. Bioactive Glass (BAG) [34], particularly the S53P4 composition, is gaining traction for high-risk fractures. BAG increases local pH and osmotic pressure, providing a bactericidal effect while stimulating osteoblast proliferation [35,36,37,38].

Lindfors et al. (2010) demonstrated successful treatment of chronic osteomyelitis using BAG S53P4, with 9 of 11 patients healing without complications at a mean follow-up of 24 months [36]. Tanner et al. (2018) further established a randomized controlled trial protocol evaluating BAG S53P4 in tibial and femoral non-unions, underscoring its growing role in high-risk fracture management [37].

Hybrid devices, such as Cal-Cemex [39], which integrate β-TCP with polymers like PMMA, are also being utilized to provide immediate structural stability in weight-bearing sites, allowing for earlier mobilization.

Beyond pure-phase β-TCP, ion-substituted forms have emerged as functionally enhanced alternatives. Strontium-substituted β-TCP (Sr-TCP) has demonstrated improved osteoblast proliferation and inhibition of osteoclast activity, making it particularly attractive in the osteoporotic environment. Magnesium-substituted β-TCP (Mg-TCP) enhances mechanical strength and promotes angiogenesis, while silicon-substituted TCP improves protein adsorption and cell adhesion. These substituted forms represent a promising next step in tailoring resorption kinetics and biological activity to patient-specific needs.

## 8. Emerging Technologies: 3D Printing and Nanotechnology

Three-dimensional printing allows for the fabrication of personalized β-TCP scaffolds that match the exact geometry of a patient’s distal tibia defect [6]. These scaffolds can be engineered with graded porosity and loaded with bioactive peptides to maximize vascular infiltration. Nanotechnology further enhances these materials by creating nanocomposites that mimic the microscopic structure of natural bone, improving cell adhesion and proliferation. 3D-printed titanium implants have demonstrated 100% osseointegration in distal tibial defects, though evidence remains insufficient to define their role versus traditional techniques [40]. Unlike permanent titanium constructs, resorbable β-TCP scaffolds are better suited for contained metaphyseal voids, avoiding stress shielding—a particular concern in osteoporotic bone.

Recent preclinical work combining PLGA, decellularized bone matrix microparticles, and magnesium hydroxide has demonstrated stage-matched regulation of inflammation, neovascularization, and osteogenesis [41]. While promising, clinical translation remains limited by fabrication complexity, and pure β-TCP continues to offer an immediately applicable solution with an established safety profile.

Despite promising preclinical results, the clinical translation of both 3D-printed β-TCP scaffolds and hybrid constructs remains limited by fabrication complexity, regulatory hurdles, and lack of large-scale clinical trials. Current evidence supports their use as adjuncts rather than replacements for established materials, and further high-quality studies are warranted to define their role in routine orthopedic practice.

## 9. Conclusions and Clinical Recommendations

The management of osteoporotic distal tibia fractures requires a multi-faceted approach. Current evidence suggests that pure β-TCP represents a promising osteoconductive option for metaphyseal void filling, with powder-type formulations demonstrating potential advantages in packing efficiency and resorption kinetics. However, the level of evidence remains largely observational, and further high-quality comparative studies are needed before definitive clinical recommendations can be made. DBM offers osteoinductive potential as a biological supplement; however, current clinical evidence remains limited by study heterogeneity, and its use should be guided by careful patient selection rather than routine application [30,35]. Clinicians should move toward a personalized selection of bone graft substitutes, utilizing high-purity powders for complex void filling and considering bioactive glass or hybrid materials for cases requiring antimicrobial or immediate mechanical support.

## Figures and Tables

**Table 2:** Comparative properties of common bone graft materials.

Property	Autograft	DBM	Powder β-TCP	Granular β-TCP	Bioactive Glass
Origin	Patient	Human donor	Synthetic	Synthetic	Synthetic
Packing Efficiency	High	Moderate	Excellent	High	Moderate
Surface Area	N/A	High	Very High	High	Moderate
Osteoinduction	High	Moderate	None	None	Osteostimulantive
Resorption Rate	Variable	Moderate	Fast/Predictable	Moderate	Moderate
Primary Use	Gold standard	Bio-enhancement	Irregular voids	Voids /Scaffold	Infection /Biostim

## Data Availability

No new data were created or analyzed in this study.

## Abbreviations

The following abbreviations are used in this manuscript:

β-TCP	ß-tricalcium phosphate
DBM	demineralized bone matrix
BAG	bioactive glass
